# Encouraging gender-inclusive acceptance of multipurpose national-identity smart cards

**DOI:** 10.1371/journal.pone.0271033

**Published:** 2022-07-22

**Authors:** Yuen Yee Yen, P. H. P. Yeow, Loo Wee Hong

**Affiliations:** 1 Faculty of Business, Multimedia University, Melaka, Melaka, Malaysia; 2 Department of Marketing, Sunway University Business School, Sunway University, Subang Jaya, Malaysia; Hankuk University of Foreign Studies, REPUBLIC OF KOREA

## Abstract

**Purpose:**

Gender-inclusive adoption of multipurpose national-identity smart cards (MNIS) is important to ensure gender equality, particularly in accessing public services offered by the card e.g. identity verification, healthcare, transit, banking, driving license, passport, etc. The aim is to study the gender differences in terms of the motivation and impediments of adopting MNIS to recommend gender-specific adoption strategies.

**Methodology:**

The research framework is based on the Unified Theory of Acceptance and Use of Technology (UTAUT) with the added constructs of perceived credibility and anxiety. The data was collected through five hundred questionnaires from Malaysia (the MNIS pioneer) and analyzed using structural equation modeling.

**Findings:**

The results show that females have significantly higher perceived credibility while males have significantly higher performance expectancy for MNIS. The correlation between performance expectancy and perceived credibility is significantly stronger among males.

**Practical implications:**

Strategies recommended to policymakers include having social messages related to MNIS utility and convenience in campaigns targeting males while alleviating concerns over security and privacy for campaigns targeting females.

**Originality/value:**

This is the first study that investigated the gender differences in adoption of MNIS by comparing the structural UTAUT models of both genders. The gender differences in MNIS adoption were explained using gender theories.

## 1. Introduction

A smart city requires extensive daily adoption of smart identity cards in public transportation, healthcare services, banking services and national security. The *“Malaysian Government Multipurpose Identity Smart Card”* (MyKad) was officially implemented in Malaysia on September 5, 2001 as one of the e-government flagship projects to transform the country into a developed nation by the year 2020 [1]. During its launch in September 2001, the Malaysian government proclaimed the MyKad as the world’s first MNIS [1]. Developed at a cost of RM276 million or approximately USD70 million, the card was originally embedded with a microchip with four main applications i.e. the national identity card (IC), driving license, passport and health information [1]. A few months after its implementation, the card was enhanced with five new applications i.e. e-cash, automated teller machine (ATM) access, Touch ‘n Go (transit application), frequent travelers’ cards and digital signatures (public key infrastructure). These applications were aimed at facilitating secure electronic transactions and improving the efficiency of e-government and private services in areas such as healthcare, banking, immigration, transit and payment services [1]. Unlike the old paper-based IC where thumbprints were visible on the surface of the card, MyKad utilizes biometric technology to encrypt a cardholder’s thumbprints in its chip [1]. The thumbprints stored in the chip are then verified electronically with the actual thumbs of the cardholders [1]. The identity verification process is therefore more accurate compared with the paper-based IC [1]. Criminals/terrorists who carry fake MyKad to cover up their real identities can be easily detected at immigration checkpoints or police roadblocks via the biometric verification [1, 2]. This MyKad feature has successfully attracted the attention of various countries worldwide. Numerous countries in Europe, Asia and North Africa have followed the initiatives of the Malaysian government and launched MNIS. For instance, Italy issued its MNIS on January 1, 2006 to store cardholders’ personal details on a microchip to facilitate electronic authentication [3]. Estonia issued its smart card in September 2007 to allow cardholders to sign digital documents [3]. Hong Kong introduced MNIS on June 23, 2003 to provide cardholders with quick library access, secure electronic transactions and immigration verification during travel [4]. In Morocco, more than two million cardholders have replaced their paper-based ICs with biometric MNIS since April 2008 to facilitate immigration control during travel [3]. All citizens of Hungary must have at least a valid passport, a photo-card driving license or the MNIS [3].

Nevertheless, after a decade of implementation, only ten percent of Malaysian cardholders have utilized their MyKad fully [1]. NRD officers experienced a hard time convincing 5.7 million cardholders nationwide to accept the MyKad [5]. Its applications were underutilized and this increases the financial burden of the Malaysian government as it costs the government RM33 (USD8.42) to produce each card or approximately RM660 (USD168.43) million a year to issue and dispose of cards [5]. The latest figure shows a total of 159,000 cards as awaiting collection, some of which had been ready for more than two years [6]. Additionally, there is a significant gender gap worldwide in terms of the adoption and access of technological services such as MNIS [7]. The biological characteristics of males and females affect technology inclusion [8], especially in countries where technological industries are dominated by males [7]. As stated by the Gender Role Theory, males and females have different expectations towards technology adoption [9]. Males are typically associated with higher self-confidence, aggressiveness and goal achievement, which are important traits for technology adoption [10]. The Stereotype Threat Theory holds similar findings that females are often stereotyped as being technologically inept. When a female is perceived to do poorly with a new technology due to negative stereotyping, she faces more mental pressure and greater resistance towards the technology [11]. Males bought and played more online games compared with females due to negative stereotyping of the latter, which steered them away from technology-driven activities [11, 12]. Statistics also confirm this, i.e. females show limited interest in technology and males use technology more often than females for entertainment (Padilla, et al., 2013). Higher prestige and social status are given to males (compared with females) for embracing technological products [13]. Females, on the other hand, are often criticized about their technological skills, which makes them feel technologically incompetent, thus driving them away from technology [14]. Gender differences exist also with regard to Web acceptance and use, where males’ Web usage is significantly higher than females due to the former’s higher perception of its usefulness [14].

Malaysian society has transformed from an underdeveloped to a strong developing economy in the past decades, with a significant number of educated middle-class citizens [15]. Despite the economic change, females’ involvement in technology is considerably lower than males [16]. Imbalanced adoption of technology like MNIS generates gender inequality as males who use the technology more than females will have a greater advantage in accessing digital services such as banking, transportation, payment, healthcare, education, social benefits, etc. thus, causing the digital and economic divide between the genders. This research attempts to address the divide through the following research questions:

1. Is there a gender difference in MNIS adoption?
2. What are the differences in motivation and impediments between genders in MNIS adoption?
3. What strategies can policymakers undertake to encourage gender-specific MNIS adoption?

This research contributes to the theories relating to the digital divide between genders. It adds on to existing theories explaining the differences in motivation and impediments between the genders in terms of adoption of technology such as MNIS. The expected practical contribution is that the results provide insights to policymakers globally on how to enhance their e-government MNIS initiative to ensure a balance of services between male and female citizens. This research contributes the sustainable development of a smart city, which features the utilization of the MNIS infrastructure for better quality of life among male and female citizens.

## 2. Literature review, research gap and objective

Gender-inclusive technology adoption is under-researched with limited studies conducted to gauge the impact of gender differences on technology adoption. For example, in the host country, Masrom’s [15] study highlighted that even though the Opposition in parliament and public interest groups raised several concerns over the difficulties of usage, privacy risks and lack of public consultation in terms of MNIS services, there is a lack of understanding on the difficulties faced by both genders in the adoption. Hanham et al. [16] studied gender differences in general technology adoption in terms of diffusion of innovation variables such as relative advantage, ease of use, visibility, result demonstrability, critical mass and use intention. The results showed that females value ease of use and visibility more than males while males had a higher preference for relative advantage, result demonstrability and perceived critical mass. Although relative advantage (performance expectancy) is applicable, the results from Hanham et al. [16] cannot be applied in the context of MNIS adoption because they did not include influences from perceived credibility, anxiety, social influence and facilitating conditions, which are crucial factors in MNIS adoption as presented in Loo et al.’s [17, 18] studies. Other research centered on gender differences in technology self-efficacy. Compared with females, males report higher self-efficacy towards technology adoption [19]. They are more confident and comfortable using technology [20] and more knowledgeable about all aspects of a technology [21]. There are several studies related to MNIS. Surveys by Zdnet Asia [12] found that the MNIS initiative is positively viewed in the UK and China. Small sample surveys by Yeow and Miller [22] and Yeow et al. [23] came to a similar conclusion among Malaysians. Loo and Yeow [24] examined the moderating effect of age towards Malaysians’ intention to use IC applications. Loo et al. [17, 18] and Yeow et al. [2] conducted larger sample size surveys and validated the constructs from the various MNIS applications. There is still a research gap, i.e. no study has addressed the gender differences in MNIS adoption. Access to MNIS services such as identification, healthcare, payment systems, education, immigration and other public and private services are added advantages for citizens as they increase efficiency and improve the quality of life. Thus, it is important that this e-government initiative is promoted equally to both male and female citizens to enhance gender inclusiveness. For this reason, the main objective is to investigate crucial factors affecting intention to use MNIS by males and females. This study will contribute towards an understanding of how males and females perceive MNIS adoption, which is fundamental for policymakers to plan gender-inclusive strategies. Additionally, it will provide insights to assist industry practitioners to design appropriate interventions for both genders. Moreover, gaining an understanding of the motivation and barriers to adoption can reduce MNIS implementation risks and provide a strong foundation for its future adoption. Theoretically, this study will fill the research gap by enhancing the original UTAUT with two new constructs i.e. perceived credibility and anxiety, to provide a conceptual view of both positive and negative factors that encourage or hinder MNIS acceptance by both genders. Additionally, it will be based on a large sample size, making the results more representative of the users.

## 3. Theoretical models and hypotheses

### 3.1 UTAUT model

**Fig 1:**
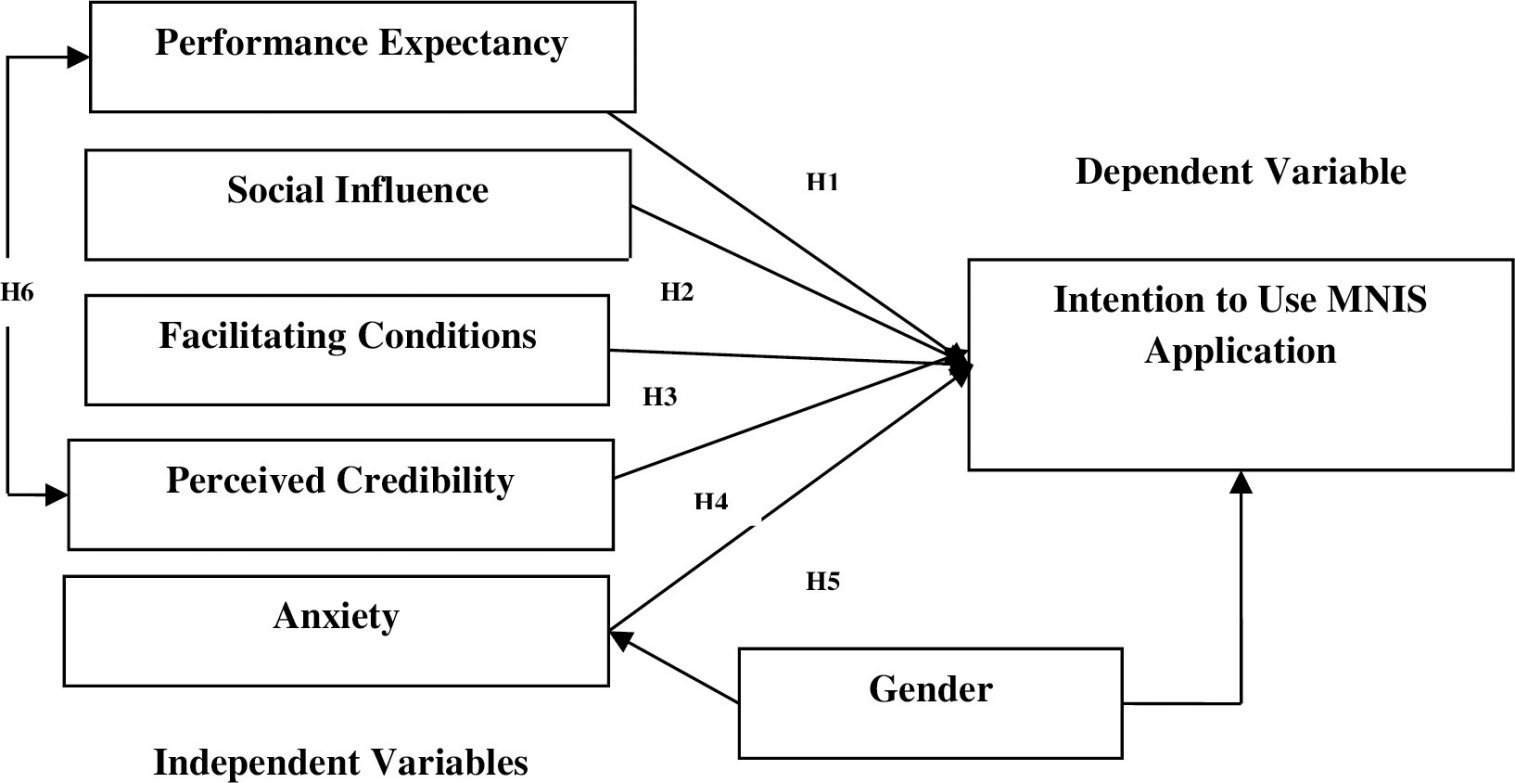
Research framework.

The UTAUT model is an advanced theoretical framework, formulated by eight established technology acceptance theories, i.e. “*Theory of Reasoned Action”*, *“Technology Acceptance Model” (TAM)*, *“Motivational Model”*, *“Theory of Planned Behaviour” (TPB)*, *“Combination TAM and TPB”*, *“Model of Personal Computer Utilization”*, *“Innovation Diffusion Theory”*, *and “Social Cognitive Theory”* [25]. The UTAUT model is a parsimonious research model that provides better insights into individual technology acceptance by encompassing the combined explanatory powers of all the established theories above [25]. All four key constructs in the original UTAUT model, i.e. performance expectancy, effort expectancy, social influence and facilitating conditions, have been carefully validated by Venkatesh et al. [25]. Although there is a new prediction framework known as UTAUT2, the hedonic motivation construct and price value construct in UTAUT2 are irrelevant to MNIS adoption as there is no hedonic aspect with the card and its cost is paid by the government. Therefore, UTAUT instead of UTAUT2 is used in this study to investigate gender adoption of MNIS. In the past few years, UTAUT had been used to examine individual acceptance of a course management software [26]. Internet banking [27, 28] and e-government services [13]. The UTAUT model together with the perceived credibility and anxiety constructs were used in prior MNIS studies by Loo et al. [17, 18] and Yeow et al. [2, 23]. Since these studies had validated the applicability of the model and constructs in the MNIS context, the present study adopted them to study gender differences in adoption, which was not addressed in the earlier studies. Effort expectancy is not included in this study because most MNIS applications require merely an IC for verification purposes, thus not requiring much effort/complexity. Therefore, the construct is excluded from this study despite being one of the constructs in the original UTAUT. Fig 1 shows the research framework of the study.

### 3.2 Intention to use

User acceptance measures a user’s intention to adopt an innovation [25, 29]. In this study, user acceptance is measured by intention to use MNIS, a highly valid measure to predict technology acceptance [19]. “Intention to use” is defined as the likelihood that a person will employ a particular technology [30]. The present study adapted this definition as the likelihood that cardholders intend, predict and plan to use MNIS in the near future for verification and identification in government and private sector transactions i.e. verification and identification done by slotting the card into a card reader. For instance, the cardholder will slot the card into a card reader in an e-kiosk or public/private service provider to verify his/her identity for transactions like immigration, banking, insurance, healthcare, etc. The card reader verifies if the information and thumbprint embedded in the card match the cardholder’s actual thumbprint. The following independent constructs are believed to have either encouraged or discouraged gender intention to use MNIS.

### 3.3 Performance expectancy

The first independent construct, performance expectancy, measures a user’s improvement in job performance following technology adoption [21, 25]. The present study adapted this definition to the MNIS context i.e. the degree to which MNIS holders believe that using MNIS applications will help them attain gains in daily life. The dimensions of “performance expectancy” are subdivided into “convenience”, “verification process”, “identity theft”, “reliability of data” and “fits into lifestyle”. Prior research found that males have significantly higher performance expectancy towards using a new technology compared with females [19]. Ong and Lai [31] and Venkatesh et al. [25] discovered that the influence of performance expectancy on behavioral intention will be much stronger for males than for females because the former will have significantly higher expectations on the performance of technical artifacts. This concurs with the Gender Role Theory that males have higher goal achievement with technology compared with females [10]. Niu et al. [32] showed significant behavioral differences in technology adoption where males have significantly higher performance expectancy in e-commerce due to their higher tendency to make a planned purchase for online products [16]. Apart from that, gender had demonstrated a significant effect on the Internet service performance expectancy where higher performance expectancy was identified among males [33]. Males are more concerned with work roles than females, thus technology’s usefulness and job productivity will be more important to males than females [33]. Similarly, in the present study, we believe that males are more concerned with performance such as efficiency and productivity in performing e-government and private services compared with females; therefore, their performance expectancies are expected to be more significant than their female counterparts.

*H1*: *Performance expectancy affects male cardholders’ intention to use MNIS more significantly than female*.

### 3.4 Social influence

The second independent construct, social influence, measures the influence of family and friends in the acceptance of a technology [34]. Similar to the “subjective norm” construct in the Theory of Reasoned Action (TRA) and TPB models [25], social influence measures the perception of social circles toward the use of a technology [30]. In the present context, social influence measures the social pressure associated with MNIS acceptance. It refers to the action invisibly exercised by other people through their opinions or orders on a user, which causes the latter to accept or resist a particular technology, e.g. intention of using MNIS applications. Within this scenario, social influence includes one’s peer group, other citizens and the government. Prior literature on gender differences and gender roles suggests that females have higher social needs than males and are thus more concerned with connecting to others as well as more likely to conform to majority opinions [15, 32]. Female workers have significantly lower need for autonomy than males, suggesting that social influences may be more important to understand female workers’ technology acceptance behavior. According to the Gender Order Theory, females have lower social prestige compared with males when it comes to positions in work, income and ownership [10]. This difference compels females to socialize more with people of the same gender in order to gain more social support. Many prior studies have found that social support strengthens a female’s practices around technology, where she is more likely to seek consultation from her peers and friends when buying and adopting a new technology [35]. Similarly, in the present study, we believe that social influence affects females more significantly than males in the adoption of MNIS technology.

*H2*: *Social influence affects female cardholders’ intention to use MNIS more significantly than male*.

### 3.5 Facilitating conditions

Facilitating conditions are another important determinant of intention to use, which measures the availability of technical infrastructure to facilitate technology acceptance [10, 25, 36]. The present study adapted this definition as the objective environment to facilitate users’ intention of using MNIS applications. The dimensions measuring “facilitating conditions” are: information on the IC is loaded into the card by default, the card as the de facto requirement to access government and private sector services, the likelihood of phasing out the old IC version, assistance from the NRD, avoidance of unpredictable fine. Facilitating conditions are important for females who are perceived to be less competent in using technology compared with males [19, 37]. They are more likely to need more resources in support of the use of a new technology compared with males [25]. In this study, facilitating conditions refer to the technical infrastructure or professional assistance that facilitates MNIS acceptance. Males have significantly higher task identification capability compared with females, thus, they are less concerned about facilitating conditions [21]. Assaker [13] reported the importance of having friendly supervisors who can provide appropriate technology-facilitating guidance to females when in need. This is supported by the Gender Role Theory, which suggests that females tend to value and respond to the opinions of their co-workers more than males, therefore they attach more importance to receiving help and assistance on the job [13]. This is consistent with Edeh et al.’s [26] findings that older females are cognizant of their lower abilities for learning and may find facilitating conditions the most effective means of acquiring new knowledge. Edeh et al. [26] also suggested that access to resources and assistance is useful in facilitating females in learning to use new technology. Similarly, in the present study, we posit that facilitating conditions have a more significant effect on females than males in MNIS adoption.

*H3*: *Facilitating conditions affect female cardholders’ intention to use MNIS more significantly than male*.

### 3.6 Perceived credibility

The next independent construct, perceived credibility, covers the security and privacy concerns associated with individual technology acceptance [25]. Perceived credibility is a psychological state in which individuals are willing to accept vulnerability due to their positive expectations of the behavior of another [27, 38]. Perceived credibility is the extent to which a potential user believes that a new technology employed by a government agency has the technical guarantees for completing transactions and transmitting sensitive information in a secure manner [35]. Since smart cards are employed by most local governments in smart cities as a payment and information exchange system to interact with many public healthcare and identification services, the perceived credibility of the smart card is a critical concern for many citizens [32]. Security concerns have been a common concern when providing public governmental services because many e-government smart-card initiatives were vulnerable to security attacks [32]. In this study, perceived credibility is defined as the security and privacy concerns relating to MNIS. Potential users in many countries (e.g. the U.S, the U.K, Taiwan, Australia, etc.) have resisted the use of smart card stemming from their fears that it is not secure. This fear is usually associated with issues of card forgery, identity theft and abuse, all of which could erode their privacy should the information embedded in the card be easily viewed by others [12, 16]. Consequently, “perceived credibility” in this study refers to user perception that the MNIS is secure (i.e. the card is difficult to forge, and therefore, safe from identity theft) and free from privacy threats (i.e. to have sensitive information divulged by a third party without the user’s permission or knowledge). Of particular relevance to the perceived credibility domain is that females are more concerned about forming and maintaining relationships with friends and willing to provide their personal details to friends [26]. Males, on the other hand, are more concerned about the privacy and security of their personal data in the context of relationships [35]. Conversely, in different circumstances, females are more concerned about the safety of using a new technology, such as credit-card misuse or fraud, compared with males [10]. According to Khoza et al. [35], female customers in e-commerce are more rational and more sensitive to risks than their male counterparts, perceived credibility on purchase intention was stronger for females than males, and female customers were more cautious and conservative while consuming a new innovation or product. Females have a higher need for perceived credibility compared with males in the adoption of new technology, which improves their attitude and thus increases intention [39]. On the basis of this argument, the following hypothesis is formed:

*H4*: *Perceived credibility affects female cardholders’ intention to use MNIS more significantly than male*.

### 3.7 Anxiety

The anxiety construct measures the anxiousness associated with the use of contemporary technology [40]. It refers to negative emotions in cognitive states evoked in actual or imaginary interaction with computer-based technology [7]. Consistent with computer-based technology, potential MNIS users typically indicate negative behavior like not using the card owing to anxiety about losing or damaging it [2, 41]. Thus, “anxiety” in this study refers to the negative emotions evoked when using MNIS applications. The dimensions measuring “anxiety” are subdivided into intimidation, hesitation and apprehension. In a recent study of citizens in China, Poland and Sweden, females reported in general a higher degree of anxiousness when it comes to technology acceptance [34]. Males are not worried when using new technology but females are afraid to use a new technology such as the smart card [34]. Males report lower levels of anxiety around technology [19], are more comfortable using technology [20] and more knowledgeable about all aspects of a technology [21]. This digital divide or “gender gap” [20] is an international phenomenon where females have higher anxiety than males towards new technology [19]. Winarno and Putra [19] posited that males being more motivated by extrinsic goals, do not perceive technology as a threat but as a means to achieve their goals. Females, on the other hand, view technology negatively during the initial contact, spend more time later in anxiety and finding ways to cope with their negative beliefs, which may influence their adoption. Similarly, in the present study, we believe that anxiety affects females more than males in the adoption of MNIS.

*H5*: *Anxiety affects female cardholders’ intention to use MNIS more significantly than male*.

Prior research found that users’ performance expectancy increased with the increase in trust towards the safety of personal information stored in a smart card [2]. Additionally, females have less perceived credibility in handling technologies compared with their male counterparts [10]. Their lower perceived credibility leads to a lower performance expectancy compared with males [29]. In contrast, males tend to report a higher level of perceived credibility and performance expectancy [27]. Similarly, in the present study, we believe that the finding applies to MNIS technology.

*H6a*: *Perceived credibility correlates with male cardholders’ performance expectancy more significantly than female*.

## 4. Method

Five hundred questionnaires were developed based on the UTAUT model, trialed in a pilot study and distributed to MNIS holders who reside or work in the MSC Malaysia. The MSC Malaysia covers large urban regions, including the Federal Territory, Cyberjaya, Putrajaya and Selangor. A copy of the questionnaire used can be found in S1 Appendix. The data collection involved conducting face-to-face recruitment, exposure and data collection with the respondents. Questionnaires were distributed to technology savvy (used multiple MNIS applications) and less technology savvy (did not use MNIS application) citizens in MSC Malaysia to conduct a holistic examination on how performance expectancy, facilitating conditions, social influence, perceived credibility and anxiety affect their intention to use MNIS. Since the NRD branches nationwide are unable to disclose the contact details of current MNIS holders due to data confidentiality, convenience sampling was applied in this study. Questionnaires were distributed to MNIS holders at government offices, supermarkets, bus stations and other public places. This study was confined within the MSC Malaysia as the majority of MNIS infrastructures are found in this Special Economic Zone. In addition, MNIS issued by NRD offices in MSC Malaysia can be loaded by a wider range of applications such as identity verification, healthcare, transit, banking, driving license and passport, compared with MNIS issued by other NRD branches in Malaysia.

A questionnaire was designed based on the theoretical framework. The items of each construct/factor in the questionnaire were derived from prior literature as described in the earlier section. The questionnaire has three sections. Section A examines respondents’ demographic background, such as age, gender, race, education level, occupation, income level and experience. Section B measures key MNIS acceptance factors: performance expectancy, social influence, facilitating conditions, perceived credibility and anxiety. Section C measures cardholders’ intention to use MNIS in the near future. Questions in section A were measured using nominal and ordinal scales while questions in sections B and C were measured using the 5-point Likert scale (strongly disagree = 1, disagree = 2, neither agree nor disagree = 3, agree = 4, strongly agree = 5).

### 4.1 Ethics statement

Ethical approval was obtained for this project from the Research Ethics Committee of the Technology Transfer Office (TTO), Multimedia University (Ethical Approval Number: PD20200677). Written consent was obtained from participants for the use of and publication of their data. A written consent statement was printed on the survey. Respondents were required to tick the written consent form before they started the survey.

## 5. Results

This study has slightly more female (264) respondents than males (236) as shown in Table 1. Almost half of the respondents are Chinese (44.6%), followed by Malay (37.4%) and Indian (18.0%). Respondents aged 18–45 years comprise 73.0%, followed by respondents above 45 years of age (27.0%).

**Table 1:** Demographics of the respondents.

		Frequency	Percent (%)
**Gender**	Male	236	47.2
	Female	264	52.8
**Ethnic Group**	Malay	187	37.4
	Chinese	223	44.6
	Indian	90	18.0
**Age**	18–45 years old	365	73.0
	Above 45 years old	135	27.0

Table 2 shows that gender differences in the intention to use MNIS are not significant (p-value = 0.31).

**Table 2:** Mean scores for intention to use MNIS.

Variables	Male	Female	t-stat (p-value)
Intention to Use MNIS	3.09	3.05	1.016 (0.31)

Note

***Significant at 0.01 level.

### 5.1 Structural Equation Modeling (SEM)

SEM is employed in this study to analyze a series of structural relationships in the path diagram [42]. AMOS software is chosen as it offers user-friendly drawing tools and graphical interfaces, which facilitate the creation and validation of structural and measurement models [42]. The five hundred collected data in this study is sufficient for SEM analysis [42]. In order to test the difference in significant paths, respondents are classified into two groups, male and female cardholders.

### 5.2 Measurement model

Confirmatory factor analysis is performed to test the extent to which all indicators in the measurement models comply with previous literature [33]. Constructs with indicator loadings below 0.7, i.e. Facilitating1, Facilitating2, Facilitating3, Facilitating4, Facilitating5, Social1, Social2, Social3, Performance4, Performance5 and Credibility4 are removed from the analysis [33]. After the deletion of the above, the confirmatory factor analysis result fits well with the data, represented by Goodness-of-Fit Index (GFI) >.95 (.966), Comparative Fit Index (CFI) >.95 (.969), Tucker Lewis Fit Index (TLI) >.95 (.954), Normed Fit Index (NFI) >.95 (.957) and Root Mean Square Error of Approximation (RMSEA) < .08 (.068) [33].

All constructs in the measurement model of male and female cardholders in Table 3 have critical ratios greater than their Mardia coefficients, indicating high data normality [33]. In addition, construct reliability of the measurement model of both genders is verified by having Cronbach’s alpha coefficient of greater than 0.6 for all constructs [43].

**Table 3:** Measurement model for male and female cardholders.

Gender	Construct	Item	Loading	Rank	Mardia Coefficient	Critical Ratio	Cronbach Alpha	AVE
Male	Performance Expectancy	Performance1	0.81	1	3.63	5.09	0.73	0.61
		Performance2	0.83	2				
		Performance3	0.48	3				
	Perceived Credibility	Credibility1	0.77	2	5.82	8.16	0.70	0.56
		Credibility2	0.46	3				
		Credibility3	0.80	1				
	Intention to Use	Intention1	0.69	1	1.42	1.99	0.60	0.61
		Intention2	0.61	2				
		Intention3	0.46	3				
Female	Performance Expectancy	Performance1	0.88	1	3.33	4.95	0.82	0.65
		Performance2	0.87	2				
		Performance3	0.62	3				
	Perceived Credibility	Credibility1	0.83	2	7.13	10.58	0.79	0.64
		Credibility2	0.59	3				
		Credibility3	0.84	1				
	Intention to Use	Intention1	0.81	1	0.75	1.11	0.78	0.73
		Intention2	0.77	2				
		Intention3	0.65	3				

Construct validity is verified in terms of convergent, content and discriminant validities (Table 4). Convergent validity is verified by Average Variance Extracted (AVE) of greater than 0.50 [33]. Content validity is verified through comprehensive literature reviews prior to the development of the research framework and hypothesis. Discriminant validity is verified by the square root of AVE greater than the inter-construct correlations [33]. None of the constructs has Pearson coefficient of greater than 0.8, which violates the “non-multicollinearity” assumption of SEM [33].

**Table 4:** Correlation matrix for male and female cardholders.

Gender	Construct	1	2	3
Male	Performance Expectancy	**0.78**		
	Perceived Credibility	0.40	**0.75**	
	Intention to Use	0.60	0.58	**0.78**
Female	Performance Expectancy	**0.81**		
	Perceived Credibility	0.35	**0.80**	
	Intention to Use	0.63	0.64	**0.85**

Note: Square root of AVE is shown in the diagonal of the correlation matrix in bold. Off diagonal are the inter-construct correlations.

### 5.3 Structural model

**Fig 2:**
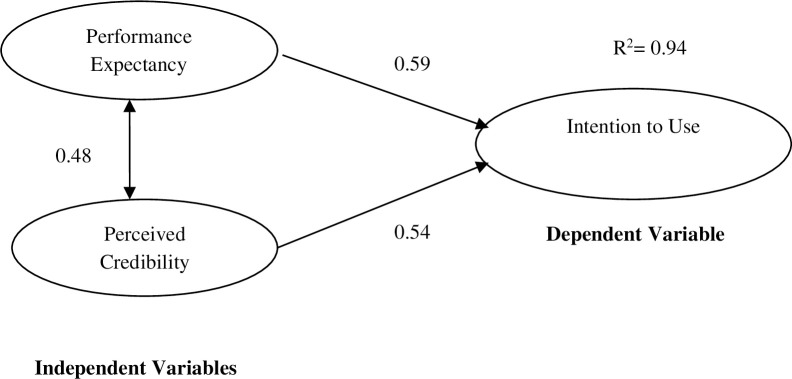
Structural model for male cardholders.

**Fig 3:**
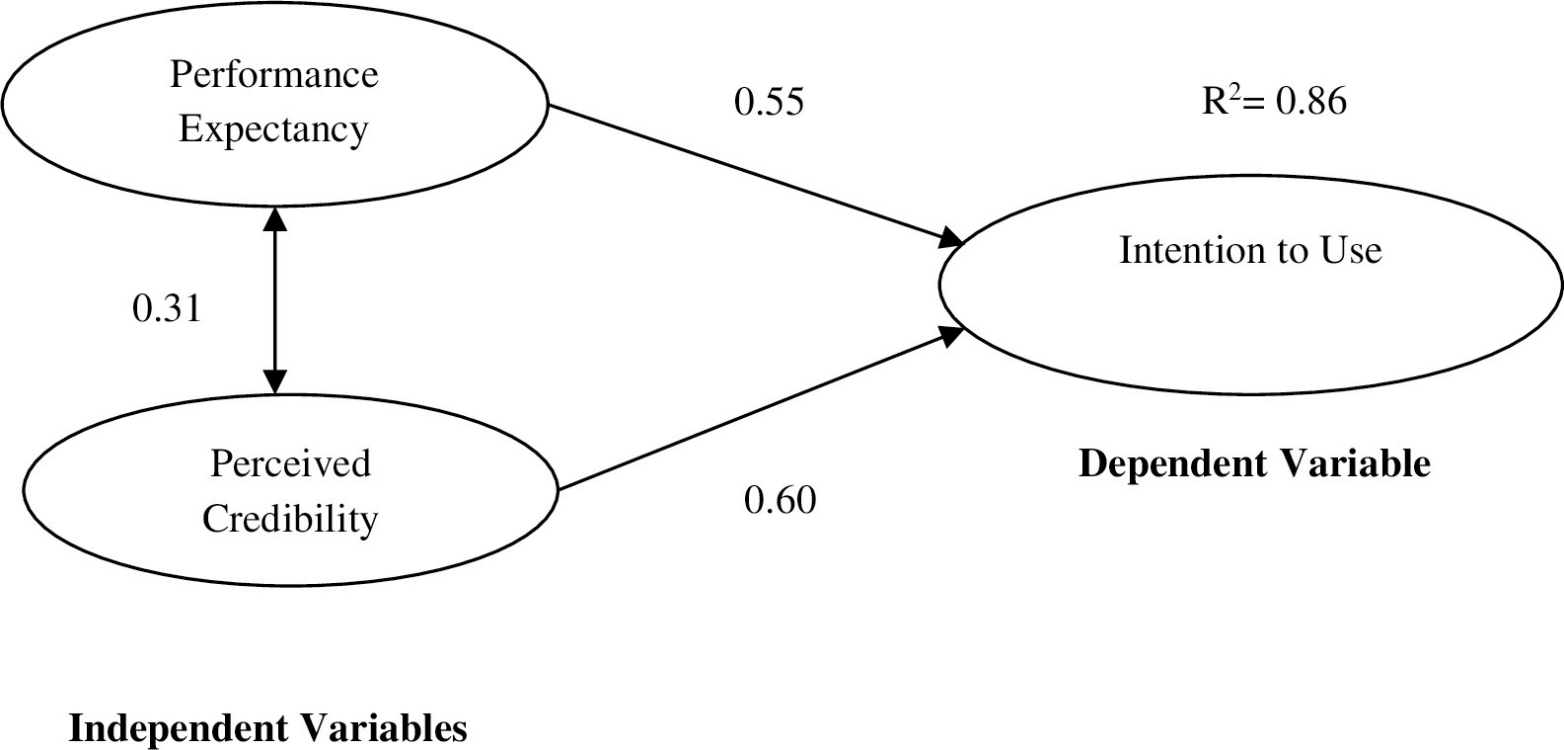
Structural model for female cardholders.

Twenty UTAUT models are drawn and compared in order to determine the parsimonious structural models for both genders. Model trimming is applied to delete Anxiety1, Anxiety2 and Anxiety3, one after another, in the process of identifying parsimonious structural models. The models for both males and females in Figs 2 and 3 exhibit acceptable goodness-of-fit represented by Goodness-of-Fit Index (GFI) >.95 (.966), Comparative Fit Index (CFI)>.95 (.969), Tucker Lewis Fit Index (TLI)>.95 (.954), Normed Fit Index (NFI)>.95 (.957) and Root Mean Square Error of Approximation (RMSEA) < .08 (.068) (Hair et al., 2006). The Browne-Cudeck Criterion (BCC) and Akaike Information Criterion (AIC) values, 122.381 and 121.522, are the lowest among all 20 drawn models, accentuating their parsimonious nature [33].

The dropping of the anxiety construct can be justified by prior studies that found that anxiety’s importance diminished with the user’s familiarity with technology [40]. Since all cardholders are familiar with the MyKad, which is an enhanced version of the paper-based identity card, anxiety about using MNIS is not relevant. Another justification is that anxiety is mediated by effort expectancy as in Venkatesh et al.’s [25] study. However, effort expectancy of MNIS is irrelevant because the cardholder’s role of presenting the card to the service provider is simple; subsequently, anxiety is irrelevant.

Male respondents view performance expectancy (path coefficient = 0.59) as the most important factor affecting MNIS acceptance while female cardholders consider perceived credibility (path coefficient = 0.59) as the most important factor affecting their acceptance of MNIS (Figs 2 and 3). Perceived credibility (path coefficient = 0.54) plays the second most important role in males’ MNIS acceptance while performance expectancy (path coefficient = 0.55) is the second most important factor for females. Perceived credibility is found to positively correlate with performance expectancy of males and females (path coefficient = 0.48 and 0.31 respectively). These two key constructs explain 94.0% of the variance in male cardholders’ and 86.0% of the variance in female cardholders’ intention to use MNIS (R^2^ = 0.94 and 0.86).

### 5.4 Multiple group analysis

A multiple-group analysis in AMOS is used to estimate the difference between genders [42]. Besides testing paths between independent and dependent constructs, the multiple-group analysis also tests the difference in path magnitude and direction across males and females. There are two main steps of multiple-group analysis [33]. The first step is to run an unconstrained model where differences in parameter estimates are allowed [33]. The next step is to run a constrained model where all parameter estimates are constrained to be equal [33]. Both models are then compared with each other [33]. The chi-square result in Table 5 reveals a significant difference across the unconstrained model and constrained model of male and female cardholders (χ ^2^ difference = 46.369 at p-value = 0.001). Both male and female cardholders share the same path models but with a significant difference in the path coefficients.

**Table 5:** Chi-square difference test.

Moderating Variable	Model	χ ^2^	Degree of freedom	p-value
Gender	Unconstrained model	91.003	48	.000***
	Constrained model	137.372	69	.000***
	χ ^2^ Difference	46.369	21	.001***

Note: ***Significant at 0.01 level.

Table 6 shows that performance expectancy has a significantly higher influence on male cardholders’ intentions to use MNIS while perceived credibility has a significantly higher influence on females. Performance expectancy is found to have a higher correlation with the perceived credibility of male cardholders compared with females.

**Table 6:** Summary of hypotheses testing.

Hypothesis	Male	Female	Hypothesis Testing
	Path Coefficient	Path Coefficient	
H1: Performance Expectancy	0.59***	0.55***	Supported
Intention to Use			
H2: Social Influence	Deleted during confirmatory factor analysis	Deleted during confirmatory factor analysis	Not Supported
Intention to Use			
H3: Facilitating Conditions	Deleted during confirmatory factor analysis	Deleted during confirmatory factor analysis	Not Supported
Intention to Use			
H4: Perceived Credibility	0.54***	0.60***	Supported
Intention to Use			
H5: Anxiety	Deleted during model trimming process	Deleted during model trimming process	Not Supported
Intention to Use			
H6: Perceived Credibility	0.48***	0.31***	Supported
Performance Expectancy			

Note

***Significant at 0.01 level

**Significant at 0.05 level.

## 6. Discussion

Different genders have different motivation and impediments towards MNIS adoption.

### 6.1 Female users

Female cardholders are more concerned about the security of using MNIS compared with their male counterparts (Table 6). They ranked perceived credibility as the most important factor affecting MNIS acceptance (Fig 3) and placed their highest concern as the security of carrying the smart card around (Credibility3, loading = 0.84, Table 3). There were 10,282 reported violence cases in Malaysia from January 2014 to January 2017, involving 2,651 male and 7,631 female victims [41]. For example, females have become major victims of purse snatching, 79% of them agree that purse snatching is a big problem [36]. The ineffectiveness of law enforcement to bring the perpetrators to justice further intensifies their security concerns to carry their MNIS on the streets [44]. In addition, high charges for MNIS replacement due to loss, i.e. RM110 (USD28.1) for first-time loss, RM310 (USD79.2) for the second time and RM1,110 (USD280.9) for third- to fifth-time losses (NRD, 2017) may also possibly make female cardholders afraid to carry their cards. MNIS forgery concern is higher among female cardholders (Credibility1, loading = 0.83, Table 3) compared with males (Credibility1, loading = 0.77, Table 3). Competence of government departments such as the NRD in utilizing the latest technology to track down fake MNIS is therefore important to promote acceptance (Backhouse & Halperin, 2008). Female cardholders also showed higher concerns over the privacy of their personal data (Credibility2, loading = 0.59, Table 3) compared with their male counterparts (Credibility2, loading = 0.46, Table 3). They are more worried about the unauthorized use of personal data and afraid that the authorities will access their personal information without their permission [7, 38]. Prior research showed similar security concerns in European countries where females have lower trust in the integrity of MNIS compared with males [7, 38].

#### 6.1.1. Implications and recommendations

The findings imply that to have a gender-inclusive adoption of the MNIS, the female need for perceived credibility should be addressed by policymakers. Since female cardholders are concerned about the security of carrying MNIS and losing their card to snatch thefts, it is recommended that cardholders should be exempted from the penalties of losing their MNIS. In order to tackle MNIS forgery, a chip embedded with the latest laser technology and stronger ultraviolet adhesive image can be deployed. The government should also ensure that some vital data in MNIS should be write-protected such as the name, IC number, citizenship status and biometric thumbprints, which cannot be replaced without destroying the card [28]. Additionally, all enforcement officers at border checkpoints, police roadblocks and airports should conduct biometric verification by scanning the physical thumbprints and comparing them with the thumbprints stored in the MNIS to identify the card’s real owner [2]. The government should target social messages at female cardholders to increase their awareness of the difficulty to forge the smart card because it is heavily encrypted with 2^128^ combinations [2, 24]. Since females place high concern over the capability of the authorities to deal fairly with their personal data, legislation should be enacted and enforced to safeguard personal information stored in MNIS. The Personal Data Protection Act in the host country only protects misuse by private companies. Misuse by public entities is excluded [2]. Amendments to the current legislation could be done to address the issue of government officers who sell the personal data of MNIS holders to unauthorized third parties e.g. by introducing stiffer penalties. Frequent internal audits should also be conducted to control this problem. The government should regulate the MNIS information collection, storage and disposal activities of government-linked and private companies. The government administration needs to carefully maintain a high level of privacy in relation to MNIS, given that the amount of personal information stored and managed by the smart card has grown rapidly in the past decades in Malaysia in line with the government’s move to attain developed-nation and smart-city goals. Thus, MNIS developers should assure all users, particularly females, that the smart card is secure and worthy of use. The government and MNIS developers should acknowledge not only the costs but also the security aspects of MNIS in implementing the smart-cities plan (Loo et al., 2009). Additionally, they should communicate the security measures to protect consumer data to all female citizens through social-marketing campaigns to alleviate the latter’s concerns.

### 6.2 Male users

Performance expectancy has a more important effect on males’ intention to adopt MNIS compared with females (Figs 2 and 3 and Table 6). Male cardholders have significantly higher expectations over the benefits and advantages they can obtain from MNIS. This concurs with Venkatesh et al.’s (2003) finding that males and females differ in their perceptions and expectations of a new technology. Males tend to be task- and achievement-oriented compared with females [26, 45, 46]. They pay significantly more attention to whether MNIS can improve their task performances or accomplish required tasks within the shortest period [25]. Convenience (Performance1, loading = 0.81, Table 3) is one of the main benefits of MNIS from the male perspective. Males are highly attracted to the accessibility and availability of applications e.g. the healthcare application that can be used in any public hospital at any time. They like MNIS applications which can fit well into their lifestyles (Performance2, loading = 0.83, Table 3). They prefer the multiple functions of identity verification, healthcare, transit, banking, driving license and passport in one card and consider it highly practical for use. Moreover, the efficiency of information verification via MNIS (Performance3, loading = 0.48, Table 3) is also an important performance expectancy feature that attracts male cardholders. MNIS can perform identity verification very quickly for many government applications through the fingerprint biometric template stored in the card, thus speeding up the processing time for immigration, banking, hospital admission, etc. MNIS also have the capability to search through remote government databases to confirm the authenticity of fingerprint biometrics through a mobile card acceptance device. Males are more likely to organize their tasks in a polychronic manner compared with females, which makes them more attracted to this efficiency advantage. Due to the male’s natural proclivity toward competitiveness (Backhouse and Halperin, 2008), performance expectancy has a more significant influence on his intention to adopt MNIS as the technology brings more tangible and practical benefits.

Male cardholders are more likely to correlate perceived credibility with performance expectancy (male path coefficient = 0.48 vs female path coefficient = 0.31, Table 6). Those who trust government agencies in handling their personal data are more likely to find MNIS useful. This concurs with Backhouse and Halperin’s [38] findings that cardholders who trust the authorities in protecting their personal information are more willing to explore new functionalities of MNIS. Therefore, enhancing males’ perceived credibility of the MNIS will lead to the increase in their performance expectancy of its application.

#### 6.2.1. Implications and recommendations

The findings imply that male users are driven by the benefits of using MNIS applications. It is recommended that policymakers convince male users by emphasizing the convenience of loading multiple applications in one card as that will reduce the number of cards in the wallet, improve productivity and make life easier [23]. The convenience of using multiple applications anytime and anywhere should be used as the main attraction to motivate them to carry their MNIS at all times. To fit into the lifestyle of male citizens, they should be provided with high-quality MNIS content and services, which can be easily accessed via fixed and mobile smart-city infrastructure and high-performance ICT. Social networks, ratings and user-feedback systems (e.g. through mobile apps) should be set up for every MNIS application, which can be a useful source of information for male users to discover the applications’ benefits and to decide whether to adopt them. Since male cardholders show high expectancy over the use of MNIS for quicker information-verification process, the government should consider installing more MNIS readers at national borders, airports, immigration offices and police stations. This will have the added benefit of making the country safer since MNIS have the ability to accurately identify terrorists/criminals through biometric verification [2, 23]. As the performance expectancy of male cardholders is closely associated with their perceived credibility of MNIS, they need to be convinced of the credibility of using the smart card before they can enjoy its benefits. Security features such as write-protection, 128-bit encryption and biometric verification should be extensively promoted through government social messages to improve trust in the card.

## 7. Conclusion and limitations

This is the first study that investigated the gender differences in adoption of MNIS by comparing the structural UTAUT models of both genders. The study found that perceived credibility and performance expectancy are crucial factors to promote gender-inclusive adoption of MNIS. Key findings such as females having significantly higher concern over perceived credibility while males have significantly higher performance expectancies over the use of MNIS provide an insight to policymakers on how to increase the adoption of MNIS using gender-specific strategies. The gender differences in MNIS adoption were explained using gender theories. This study is useful for policymakers in countries that are considering or which have already implemented MNIS such as the U.S., Germany, Italy, Estonia, Hong Kong and Morocco. Based on the findings, policymakers will be able to define strategies and produce differentiated value propositions to promote gender-inclusive adoption of MNIS. If a MNIS campaign targets male users, the promoted application should be utilitarian to ensure higher probability of use. On the other hand, if it targets females it should be focused on concerns about security and/or privacy as a way of improving trust and therefore leading to intention towards adopting the MNIS application. In particular, these gender-inclusive strategies could be accompanied by the presence of comprehensive MNIS policies at federal and local governments as well as good MNIS security and functional qualities.

### 7.1 Limitations

The data was collected from respondents in the MSC Malaysia, which limits the generalizability of results. Future research could examine crucial factors affecting MNIS acceptance in rural regions in Malaysia. Other cultural factors such as high-context versus low-context and polychronic versus monochronic (Hall, 1989) can be used to compare the perceived credibility and performance expectancy of cardholders in different countries. Performance expectancy and perceived credibility of white-collar and blue-collar cardholders can also be determined to have a better understanding of their specific needs. This research investigated gender differences in Malaysians’ intentions to use MNIS. Cultural differences may affect the applicability of the findings in other countries. The extent to which the results can be employed in other countries is unknown unless the study is replicated in those countries.

## Supporting information

S1 Datasets (XLSX)

S1 Appendix Questionnaire instrument. (DOCX)
