# Supplementary material for: Encouraging gender-inclusive acceptance of multipurpose national-identity smart cards
Source: PLoS One. 2022 Jul 22;17(7):e0271033. doi: 10.1371/journal.pone.0271033 (PMC9307195; doi:10.1371/journal.pone.0271033)
Supplement: S1 Appendix — (DOCX) [file pone.0271033.s002.docx]

**Appendix A: Questionnaire Instrument**

**Survey Questionnaire on User Acceptance of the Malaysian Government’s Multipurpose Smart Card- MNIS**

The objective of this survey is to measure users’ intention to use MNIS’s applications. Your co-operation in completing this survey questionnaire as accurately as possible is much appreciated. All information provided will be kept confidential.

**Section A: Demographic Information**

Please circle the number which best represents your response.

1. Age group

| 1 | 18 – 24 years old | 2 | 25-45 years old | 3 | 46-63 years old | 4 | 64 years old and above |
| --- | --- | --- | --- | --- | --- | --- | --- |

2. Gender

| 1 | Male | 2 | Female |
| --- | --- | --- | --- |

3. Race

| 1 | Malay | 2 | Chinese | 3 | Indian | 4 | Others (please state)………… |
| --- | --- | --- | --- | --- | --- | --- | --- |

4. Highest level of education

| 1 | Primary | 2 | Secondary | 3 | STPM | 4 | Diploma |
| --- | --- | --- | --- | --- | --- | --- | --- |
| 5 | Degree | 6 | Master Degree | 7 | PhD level | 8 | Others (please state)…………… |

5. Nature of occupation

| 1 | Student | 2 | Housewife | 3 | Self-employed | 4 | Businessman |
| --- | --- | --- | --- | --- | --- | --- | --- |
| 5 | Education | 6 | Engineering | 7 | Management | 8 | Marketing |
| 9 | Retiree | 10 | HealthCare | 11 | Information Technology | 12 | Human Resource |
| 13 | Banking and Finance | 14 | Maintenance | 15 | Other |  |  |

6. Monthly income level

| 1 | RM0-RM2000 | 2 | RM2001-RM4,000 | 3 | RM4001-  RM6000 | 4 | RM6,001-  RM8,000 | 5 | Over RM8,000 |
| --- | --- | --- | --- | --- | --- | --- | --- | --- | --- |

7. Frequency of using MNIS’s IC application (Experience)

Please circle the number that best describes your actual use of the applications.

Per 10 times, how many times you use the MNIS IC (your card is slotted into the card reader for identification) when required by respective authorities.

| **MNIS’s application** | **Actual Use(times)** | | | | | | | | | | |
| --- | --- | --- | --- | --- | --- | --- | --- | --- | --- | --- | --- |
| Identity Card (IC) | 0 | 1 | 2 | 3 | 4 | 5 | 6 | 7 | 8 | 9 | 10 |

**Section B: Factors Affecting Intention to Use MNIS’s Applications**

Please circle your most appropriate response which best describes your agreement or disagreement on the factors that affect your intention to use MNIS’s applications.

Scale: 1=strongly disagree, 2=disagree, 3=neither agree nor disagree, 4=agree, 5=strongly agree

| **1** | **Performance expectancy** |  |  |  |  |  |
| --- | --- | --- | --- | --- | --- | --- |
| Performance1 | Using MNIS’s applications are convenient. | 1 | 2 | 3 | 4 | 5 |
| Performance2 | MNIS’s applications can fit well into my lifestyle | 1 | 2 | 3 | 4 | 5 |
| Performance3 | Using MNIS’s applications allow quicker verification. | 1 | 2 | 3 | 4 | 5 |
| Performance4 | MNIS’s applications enhance reliability of my personal data. | 1 | 2 | 3 | 4 | 5 |
| Performance5 | Using MNIS’s applications can minimize identity theft. | 1 | 2 | 3 | 4 | 5 |
| **2** | **Social Influence** |  |  |  |  |  |
| Social1 | My peer group^^[[1]](#footnote-1)^^ affects my intention to use MNIS’s applications. | 1 | 2 | 3 | 4 | 5 |
| Social2 | Most Malaysians have applied for MNIS. | 1 | 2 | 3 | 4 | 5 |
| Social3 | The Malaysian government’s encouragement affects my intention to use MNIS’s applications. | 1 | 2 | 3 | 4 | 5 |
| **3** | **Facilitating Conditions** |  |  |  |  |  |
| Facilitating1 | Old IC is likely to be phased out soon. | 1 | 2 | 3 | 4 | 5 |
| Facilitating2 | IC is loaded automatically into MNIS (IC is a primary function of MNIS). | 1 | 2 | 3 | 4 | 5 |
| Facilitating3 | MNIS is a de facto requirement^^[[2]](#footnote-2)^^ to access government and private sector applications. | 1 | 2 | 3 | 4 | 5 |
| Facilitating4 | I can obtain assistance from the National Registration Department (NRD)’s staff if I have any inquiry about MNIS’s applications. | 1 | 2 | 3 | 4 | 5 |
| Facilitating5 | I apply for MNIS’s applications mainly to avoid unpredictable punishment (e.g. fine). | 1 | 2 | 3 | 4 | 5 |
| **4** | **Perceived Credibility (Security and Privacy)** |  |  |  |  |  |
| Credibility1 | MNIS’s applications are difficult to forge. | 1 | 2 | 3 | 4 | 5 |
| Credibility2 | Using MNIS would erode my privacy^^[[3]](#footnote-3)^^. | 1 | 2 | 3 | 4 | 5 |
| Credibility3 | Using MNIS is secure. | 1 | 2 | 3 | 4 | 5 |
| Credibility4 | MNIS’s applications are able to reduce identity theft^^[[4]](#footnote-4)^^. | 1 | 2 | 3 | 4 | 5 |
| **5** | **Anxiety** |  |  |  |  |  |
| Anxiety1 | I feel apprehensive^^[[5]](#footnote-5)^^ about using MNIS’s applications (e.g. fear of losing my MNIS). | 1 | 2 | 3 | 4 | 5 |
| Anxiety2 | MNIS’s applications are intimidating^^[[6]](#footnote-6)^^ to me (e.g. no confidence in MNIS’s security features). | 1 | 2 | 3 | 4 | 5 |
| Anxiety3 | I hesitate^^[[7]](#footnote-7)^^ to use MNIS’s applications (e.g. fear that my MNIS may be damaged due to extensive use). | 1 | 2 | 3 | 4 | 5 |

**Section C: Intention to Use MNIS’s Applications**

Please circle the number that best describes your level of intention to use MNIS’s applications.

Scale: 1=strongly disagree, 2=disagree, 3=neither agree nor disagree, 4=agree, 5=strongly agree

| **6** | **Intention to Use** |  |  |  |  |  |
| --- | --- | --- | --- | --- | --- | --- |
| Intention1 | I intend (expect) to use MNIS’s applications in the near future. | 1 | 2 | 3 | 4 | 5 |
| Intention2 | I predict I will use MNIS’s applications in the near future. | 1 | 2 | 3 | 4 | 5 |
| Intention3 | I plan (will make arrangement) to use MNIS’s applications in the near future. | 1 | 2 | 3 | 4 | 5 |

1. Peer group: refers to a group of people who are of the same age, social class, etc. as yourself. [↑](#footnote-ref-1)
2. De facto: really existing although not legally stated to exist [↑](#footnote-ref-2)
3. Erode privacy: easy access to a cardholder’s personal information (e.g. bank account information, etc.) even without his/her permission. [↑](#footnote-ref-3)
4. Identity theft: any crime in which someone steals personal information about and belonging to another person, for example bank account number or driving license number, and uses this information to deceive other people and get money or goods. [↑](#footnote-ref-4)
5. Apprehensive: worried or nervous about something that you are going to do, or about the future [↑](#footnote-ref-5)
6. Intimidating: making you feel worried and not confident [↑](#footnote-ref-6)
7. Hesitate: to pause before saying or doing something because you are nervous or not sure [↑](#footnote-ref-7)
